# Joint Corticosteroid Injection Associated With Higher Physical Therapy Compliance in Knee Osteoarthritis

**DOI:** 10.7759/cureus.16403

**Published:** 2021-07-15

**Authors:** Eduardo Mantovani Cardoso, Dominique Feterman Jimenez, Chia-Ling Kuo, Jason Jacob

**Affiliations:** 1 Internal Medicine, University of Connecticut School of Medicine, Farmington, USA; 2 Biostatistics and Epidemiology, University of Connecticut School of Medicine, Farmington, USA; 3 Internal Medicine, Hartford Hospital, Hartford, USA

**Keywords:** physical therapy, knee corticosteroid injection, knee injection, koa, knee osteoarthritis, healthcare disparities

## Abstract

Background

Knee corticosteroid injection (KCSI) and physical therapy (PT) are two efficacious treatments for knee osteoarthritis (KOA). However, poor adherence to PT in resource-limited communities might limit its effectiveness. KCSI prior to PT might improve adherence in this population.

Methodology

This was a retrospective cohort analysis of patients referred to PT for KOA from January 01, 2018 to December 31, 2019 from an adult primary care resident clinic in Hartford, Connecticut, USA. Patients were divided into two groups, namely, those who had a KCSI around the time of the referral versus those who did not. PT adherence was evaluated in both groups.

Results

A total of 143 patients referred to PT were selected, and 11 patients were excluded. In total, 38/132 patients had a KCSI within a four-month window of the PT referral. Patients were mostly Hispanic (no injection 79.8% vs. injection 78.9%) females (80.9% vs. 71.1%), the average age was in the 60s, and over 90% were insured by either Medicaid or Medicare. In the injection group, 18/38 patients completed at least one PT visit (47.4%) versus 21/94 patients (22.3%) in the noninjection group. The odds ratio of undergoing PT was 1.38 (95% confidence interval [CI] = 1.14-1.69; p = 0.002), and the rate ratio of PT visits was 2.50 (95% CI = 1.82-3.42; p = 1.36 × 10^-8^), both adjusted for age, sex, and severity. Among those who attended at least one session, the mean number of PT visits was 5.4 in both injection and noninjection groups (median 5 versus 4).

Conclusions

In a predominantly Hispanic patient population, those who underwent KCSI were more likely to undergo PT and, as a group, attend more sessions.

## Introduction

Knee osteoarthritis (KOA) is the most common chronic articular disease, and its prevalence has doubled since the mid-20th century. It affects 16% of the adult population over 50 years of age in the post-industrial era [1]. It has been predicted that 14 million people suffered from symptomatic KOA in the first decade of the 21st century [1,2]. Moreover, the increase in the prevalence of obesity among Hispanics has been associated with an uptrend in symptomatic KOA prevalence [2]. The cost of KOA was estimated to be 34 billion dollars yearly in a study of Medicare beneficiaries in 2014 alone [3,4], constituting a common cause of disability worldwide, with over 17 million years lived with disability [5,6].

The treatment of this condition is categorized into nonpharmacological, pharmacological, and surgical options. Specifically, two common evidence-based modalities of treatment used by providers include physical therapy (PT) and knee corticosteroid injection (KCSI) [7,8]. One of the limitations of PT is adherence, which is thought to be multifactorial [9,10]. A strong factor associated with PT compliance is pain control [9], which is known to be suboptimal in the Hispanic population for multiple reasons, including medication noncompliance, barriers to care, and poor pain coping techniques [11,12].

The primary objective of this study was to evaluate if sequential therapy of KCSI followed by PT can increase PT adherence. A special interest exists in regards to adherence to PT in underserved populations such as, in our practice, Hispanic Americans. We aim to evaluate compliance in terms of undergoing any PT sessions and the number of sessions performed. Additionally, the secondary objective of this study was to evaluate if baseline characteristics predict PT adherence.

## Materials and methods

Design

We conducted a retrospective, observational, cohort analysis based on the electronic medical records of a primary care patient panel of adult patients in a resident clinic that also performs musculoskeletal injections, in Hartford, Connecticut. This study was approved by the local Institutional Review Board (HHC-IRB, HHC-2020-0137), in accordance with the 1964 Helsinki declaration of ethical standards in clinical research.

Diagnostic criteria

The diagnosis of KOA was established by the International Classification of Diseases (ICD) code documentation of KOA in the PT referral information, when available. If the diagnosis was not available and the ICD code suggested any type of knee pathology, chart review was conducted and patients were included based on the American College of Rheumatology criteria for KOA [13].

Inclusion criteria

Patients who were referred to PT for KOA (according to the diagnostic criteria) between January 01, 2018 and December 31, 2019 were included. These patients were divided into two groups: those who had a knee joint injection for KOA around the time of the PT referral (two months before or after the referral) and those who did not have a knee joint injection.

Exclusion criteria

Patients who had a knee injection within the two-year study period outside of the established time frame of KCSI and PT referral were excluded. Patients who met the criteria but had a previous knee fracture or knee joint replacement were excluded. Additionally, patients who had PT before the KCSI or started PT eight weeks after the injection were excluded, as detailed in the outcomes session.

Outcomes

**Figure 1:**
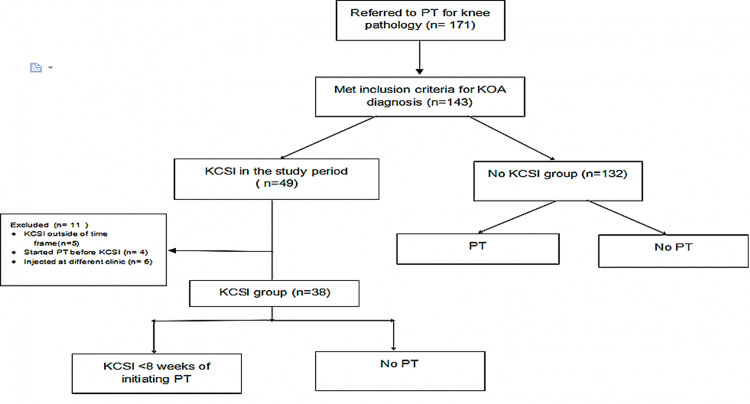
Inclusion and exclusion criteria flow sheet. KOA: knee osteoarthritis; KCSI: knee corticosteroid injection; PT: physical therapy

The assessed outcomes included the patient’s attendance to at least one PT session versus none and the number of visits completed with PT. To assess the effectiveness of sequential KCSI and PT, we excluded the patients who underwent PT either before receiving the KCSI or if they started PT eight weeks after the injection. Figure 1 details the inclusion and exclusion criteria flow sheet.

Baseline characteristics

Baseline characteristics such as age, sex, ethnicity, weight, and insurance were collected based on the chart review. Weight was used instead of body mass index (BMI) due to greater availability in our records. In addition, we evaluated KOA severity according to the radiology report using the most severe term mentioned, ranging from normal, mild, moderate, and severe. The radiology assessments were performed by board-certified radiologists without any relationship with or knowledge of the current study. The severity assessment was a rough assessment to evaluate for possible confounders.

Knee corticosteroid injection

The KCSI was performed by a proficient internist with a special interest in musculoskeletal pathology at the resident primary care clinic. The patients were injected a mix of 1% lidocaine and triamcinolone on the affected knee.

Physical therapy protocol

The PT referral was made by the primary care doctor for the treatment of the affected knee, and the treatment protocol was established by physical therapists delivering care to the patient in the same hospital system. All patients received in-person supervised therapies that may or not have been complemented by home exercises. Physical therapists established the required number of sessions and there were different therapists providing care. Patients insured by Medicaid through the state of Connecticut had no co-pays for PT.

Statistics

Statistical analysis was conducted with Fisher’s exact tests for associations between categorical variables and the group of joint injections (joint injection vs. no joint injection). Two-sample independent t-tests were performed to compare continuous baseline characteristics between the groups of joint injection. A logistic regression model was used to link the binary outcome of PT visit (yes/no) to joint injection (joint injection vs. no joint injection), with adjustment for unbalanced baseline characteristics between the two joint injection groups. The number of visits with PT (including zero visits) was modeled for the association with joint injection using a Poisson regression model after adjusting for unbalanced baseline characteristics. P-values smaller than 5% were considered statistically significant. All statistical analyses were performed in R version 4.0.5.

In the posthoc power analysis, 38 samples in the injection group and 94 samples in the noninjection group allowed us to detect a 25% difference in the proportion of PT visits between the two groups by a two-sample proportion z-test with 79% power at the 5% significance level, assuming the prevalence in the noninjection group is 25%, and a higher prevalence is expected in the injection group.

## Results

A total of 143 patients referred to PT for KOA were selected. Eleven patients were excluded from the injection group. Of those, five patients had KCSI outside of the time frame, four had started PT before the KCSI, and six were injected at different practices (orthopedics and rheumatology), with an overlap between patients who had KCSI at other services and outside the time frame for PT. Of the remaining 132 patients, 38 underwent joint injection in the time frame established for the study, and 94 did not have a knee joint injection.

Baseline characteristics are outlined in Table 1. None of the between-group differences were statistically significant (p > 0.05). Patients were mostly Hispanic (79.8% vs. 78.9%) females (80.9% vs. 71.1%), and had a mean age in the sixties (62.9 vs. 67.5) in the noninjection and injection groups, respectively. Over 90% of patients had either Medicare or Medicaid as their primary payer source, with more Medicare recipients in the injection group (52.4% ) than in the noninjection group (41.5%). The mean weight was 90.4 kg in the noninjection group compared to 88.8 kg in the injection group. Patients in the injection group had more radiographically severe KOA than those in the noninjection group (percentage of severe cases 35.5% vs. 13.8%).

**Table 1:** Baseline characteristics of both groups. SD: standard deviation

Demographics	No injection (n = 94)	Injection (n = 38)	P-value
Age (mean ± SD)	62.9 ± 10.8	67.5 ± 12.8	0.056
Sex (Female)	76 (80.9%)	27 (71.1%)	0.249
Ethnicity			0.088
Hispanic or Latino	75 (79.8%)	30 (78.9%)	
White or Caucasian	9 (9.6%)	2 (5.3%)	
Black or African American	9 (9.6%)	2 (5.3%)	
Other	1 (1.1%)	4 (10.5%)	
Insurance			0.552
Medicare	39 (41.5%)	19 (52.4%)	
Medicaid	48 (51.1%)	18 (45.2%)	
Other	7 (7.4%)	1 (0%)	
Weight (kg) (mean ± SD)	90.4 ± 28.9	88.8 ± 26.6	0.765
X-ray severity			0.056
Normal	3 (4.6%)	0 (0%)	
Mild	36 (55.4%)	11 (35.5%)	
Moderate	17 (26.2%)	9 (29.0%)	
Severe	9 (13.8%)	11 (35.5%)	

In the injection group, 18 out of 38 patients attended at least one session of PT (47.4%). Of those who underwent PT, the mean number of visits was 5.4 (median 5). In the noninjection group, 21 out of 94 patients who were referred underwent PT (22.3%), with a mean number of visits of 5.4 (median 4).

Patients with knee injections (47.4%) were more likely to undergo PT than patients without knee injections (22.3%) (p = 0.006). The odds ratio of undergoing PT on comparing patients with knee injections to patients without knee injections was 1.28 (95% confidence interval [CI] = 1.14-1.37; p = 0.004) with no adjustment versus 1.38 (95% CI = 1.14-1.69; p = 0.002), adjusted for age, sex, and severity (ordinal coding: 0 for normal, 1 for mild, 2 for moderate, and 3 for severe). The rate ratio of PT visits on comparing patients with knee injections to patients without knee injections was 2.10 (95% CI = 1.60-2.76; p = 7.13 × 10^-8^) versus 2.50 (95% CI = 1.82-3.42; p = 1.36 × 10^-8^), adjusted for age, sex, and severity. Of note, after adjusting for age and sex, severe radiographic KOA was associated with a lower likelihood of undergoing PT with an odds ratio of 0.84 (95% CI = 0.75-0.94; p = 0.002) with a 16% decreased likelihood from each progressing level from normal to severe. In addition, the rate ratio of visits for severe KOA was 0.52 (95% CI = 0.42-0.63; p = 1.02 × 10^-9^), with a 48% reduction in visits for each progressive KOA severity level.

## Discussion

An association between sequential KCSI and compliance with PT was noted in a population of mostly underserved Hispanic females. Patients who underwent a joint injection were more likely (47.4% vs. 22.3%; p = 0.006) to adhere to the PT referral and had, as a group, 2.5 times more visits based on an adjusted rate ratio of 2.5 (p = 1.36 × 10^-8^). Overall, compliance with the PT referral was mostly low and the mean number of visits for those who attended at least one session was approximately five in either group, which is less than what was reported to be effective in prior randomized clinical trials (RCTs) [14,15].

Compared to the literature, the rate of PT compliance was previously reported to be around 70-90% [9,10,15]. However, it is worth noting that these studies did not consider the starting point of a physician referral as we did, but instead included patients who initiated a PT treatment plan as part of a study enrollment criteria. To date, no study has reported the rate of compliance with a physician referral for PT, which is a broader concept that considers the time frame during which a patient was counseled regarding PT versus being already enrolled in a clinical trial. This can have implications regarding effectiveness when it comes to access to evidence-based care in underserved communities.

Several factors can affect PT compliance such as low baseline levels of physical activity, psychiatric comorbidities, social support system, and pain levels. However, data regarding the contribution of these factors are scarce among ethnic and racial minorities, such as Hispanics. Additionally, pain control is a strong contributory factor in PT compliance [9,10]. In this setting, it is worth noting that Hispanics have a higher rate of pharmacological noncompliance overall [11] and higher reported pain severity compared to other ethnic groups [12]. Additionally, compared to non-Hispanic participants, Hispanic patients are more likely to be unemployed or disabled and less likely to have private insurance [16]. The relationship between KCSI and PT adherence is under-studied, with the only data available from a Danish RCT [15], which did not show an association between prior injection and PT compliance.

There are several similarities as well as differences in our study compared to the available literature. The mean age, sex, and weight reported in our study are in line with previous reports on KOA. Osteoarthritis affects mostly obese females in the sixth decade of life [2,6]. An important distinction from known KOA prevalence pertains to race/ethnicity. Most patients with KOA in the United States are non-Hispanic whites (NHW); however, in our study around 80% were Hispanics, followed by Blacks as the second most prevalent group. NHWs were a minority in this data [2,6]. Additionally, regarding socioeconomic status, it can be inferred that the patients in this study were mostly underserved; over 90% of patients in both groups had government insurance with a higher percentage of Medicaid beneficiaries. It is worth reiterating that lack of financial support affects PT compliance. [9,10,16]

Regarding KOA treatment, recent guidelines published in 2019 by the American College of Rheumatology addressed several different modalities. Of interest, both PT and KCSI are strongly recommended by these guidelines [7]. In evaluating treatment options, a recent RCT compared PT (hands-on, passive movement followed by reinforcing exercises) and KCSI for KOA in the Veterans Health Administration. PT was found to provide a higher degree of pain relief and functionality gain, based on a validated score, in one year compared to KCSI. Of note, this trial showed a considerably positive effect of KCSI than previously reported [14].

Considering these results, strategies to increase PT compliance might be beneficial for achieving better outcomes in KOA. In our patient population, in which compliance with pharmacotherapy is low [11], the possibility of sequential therapy with a joint injection leading to symptomatic relief which would, in turn, potentially increase PT compliance, as shown by our results, is promising. The theoretical basis for this hypothesis is that anti-inflammatory therapy might be beneficial in PT by providing a synergistic effect due to decreased pain and increased patient capacity of mobilization [17,18]. Interestingly, this was tested in a Danish RCT [15], which showed no benefit of adding KCSI before PT, both in terms of compliance and the mean number of sessions. However, this study had several limitations. First, the procedure that was performed included draining the joint effusion and injecting saline mixed with lidocaine. This intervention alone might have helped PT compliance due to a possible decrease in inflammation from joint drainage and analgesia as joint effusions are associated with arthrogenic muscle inhibition and quadriceps avoidance gait patterns [17,18].

Additionally, it seems that the generalizability of these results from the Danish RCT is limited as patients were compliant with a very prolonged course of PT treatment (70% compliance with 36 sessions of PT). This does not reflect the reality of effective care delivery in the United States, especially among underserved patients. Based on these reasons, we do not think that this RCT settles the question of sequential therapy with KCSI and PT, particularly in populations with lower PT compliance.

Two main hypotheses can explain the association of KCSI and PT compliance found in this study. First, patients who received the injection might have had better pain control, which is a strong factor for PT adherence. This might be particularly important in our patient population of mostly Hispanics due to bypassing the poor medication adherence as one dose therapy can provide relief for potentially weeks to months [8]. The second potential explanation is that patients who received a KCSI had a separate visit for the procedure and were potentially counseled to a greater extent. Furthermore, we cannot exclude nor account for the possibility that patients who were compliant with KCSI might be more likely to be compliant with interventions. Lastly, due to the nature of this study and its limitations, replication studies are needed to confirm our findings.

Our study has several limitations. First, this was a single-center study that captures the reality of our practice and patient population. Moreover, it was observational and retrospective, which would, at best, allow hypothesis generation. We did not evaluate the level of pain or mobility before PT to accurately associate it with the increased adherence, nor did we analyze other barriers to PT that might be present as confounding factors. Additionally, weight was used instead of BMI due to greater data availability, which only allows us to speculate based on extrapolated anthropometric data that the BMI would be above 30 [19]. This may or not be adequate for our patient population. In regards to the inclusion and exclusion criteria, the time frame from the PT referral was selected to single out instances in which KCSI was followed by PT, excluding a small number of patients. However, our study has important strengths as it represents the real-life clinical practice and provides insights into an underserved population that is known to have more barriers to care and might be more difficult to study. In terms of the generalizability of our findings, there are some considerations to be made. Although our sample is representative of the KOA population in terms of age, sex, and weight, as it comprised mostly Hispanics, our findings may not be generalizable to other populations. As it was previously discussed, ethnicity, among other factors, might affect pharmacological adherence and pain control.

## Conclusions

While our findings might need to be replicated in a more controlled environment, ideally an RCT, they provide important insights into factors that can impact PT compliance in a population that has been historically underserved. This study should serve as a basis to further investigate the effectiveness of KCSI to improve compliance and pain management among Hispanics. Additionally, further studies examining the concepts of sequential therapy with KCSI followed by PT and of compliance with the referral for PT (initiating PT) and barriers to PT access in underserved communities are required.
